# Altered lipid metabolism in APC-driven colorectal cancer: the potential for therapeutic intervention

**DOI:** 10.3389/fonc.2024.1343061

**Published:** 2024-03-25

**Authors:** Courtney O. Kelson, Yekaterina Y. Zaytseva

**Affiliations:** ^1^ Department of Toxicology and Cancer Biology, University of Kentucky, Lexington, KY, United States; ^2^ Markey Cancer Center, University of Kentucky, Lexington, KY, United States

**Keywords:** *APC* gene, APC-mediated signaling, colorectal cancer, lipids, lipid metabolism

## Abstract

Altered lipid metabolism is a well-recognized feature of solid cancers, including colorectal cancer. In colorectal cancer, upregulation of lipid metabolism contributes to initiation, progression, and metastasis; thus, aberrant lipid metabolism contributes to a poor patient outcome. The inactivating mutation of *APC*, a vital tumor suppressor in the Wnt signaling pathway, is a key event that occurs early in the majority of colorectal cancer cases. The potential crosstalk between lipid metabolism and APC-driven colorectal cancer is poorly understood. This review collectively highlights and summarizes the limited understanding between mutations in *APC* and the upregulation of Wnt/beta-catenin signaling and lipid metabolism. The interconnection between *APC* inactivation and aberrant lipid metabolism activates Wnt/beta-catenin signaling which causes transcriptome, epigenetic, and microbiome changes to promote colorectal cancer initiation and progression. Furthermore, the downstream effects of this collaborative effort between aberrant Wnt/beta-catenin signaling and lipid metabolism are enhanced stemness, cellular proliferation, prooncogenic signaling, and survival. Understanding the mechanistic link between *APC* inactivation and alterations in lipid metabolism may foster identification of new therapeutic targets to enable development of more efficacious strategies for prevention and/or treatment of colorectal cancer.

## 1 Introduction

Colorectal cancer (CRC) is second only to lung cancer in regard to global cancer mortality for men and women combined (1). Colorectal carcinomas develop through a series of histopathological changes caused by mutations in oncogenes and tumor suppressor genes (2). Epigenetic changes, particularly aberrant DNA methylation, also contribute to pathogenesis and heterogeneity of colorectal cancer (3, 4). Mutation-driven epigenetic abnormalities of DNA methylation at the promoter CpG islands (CGIs) of genes controlling stemness, differentiation, and senescence pathways are important drivers of oncogenic transformation (5). In addition, the epigenome can influence the accumulation of somatic mutations which further promotes development of cancer (4).

The adenomatous polyposis coli (*APC*) gene is a crucial tumor suppressor and loss of *APC* function triggers the cascade of events that eventually leads to malignant transformation (6). *APC* mutations contribute to 80–85% of initiating events in sporadic CRC (7). These mutagenic modifications in *APC* can present as either germline or somatic mutations. Germline mutations in the *APC* gene result in familial adenomatous polyposis (FAP), an autosomal dominant inherited condition leading to development of multiple intestinal polyps and CRC (7, 8). Somatic *APC* mutations occur in 80% of sporadic CRCs and loss of heterozygosity (LOH) of chromosome 5q occurs in 30% to 40% of cases (7, 9). CRC appears to develop via several pathways such as chromosome instability (CIN), microsatellite instability (MSI), and serrated neoplasia pathways (2). The CIN pathway is observed in 65-70% of sporadic CRC and is associated with high *APC* mutations (approximately 80% of cases). MSI is observed in 12-15% of sporadic CRC and usually has high levels of methylation at the CGIs, but low frequency of *APC* mutations (2, 10). *APC* mutation is also less common in the formation of serrated adenocarcinomas via the serrated pathway, which accounts for 10% of all CRCs (11). In the absence of mutations in *APC*, activation of the Wnt pathway occurs via mutation of other genes or DNA methylation changes of the upstream Wnt antagonists (12).

*APC* mutations play an important role in activation of the Wnt/β-catenin signaling complex (13). Notably, more than 90% of CRCs have mutations that activate the Wnt pathway and over 80% comprise mutations in *APC* (14). The Wnt/β-catenin pathway regulates at least 80 target genes (15); is essential for cancer cell proliferation and survival; contributes to cancer cell polarity, stemness maintenance, migration, and invasion (16); supports a cancer-favorable tumor microenvironment; and dampens antitumor immune responses (16, 17).

**Figure 1:**
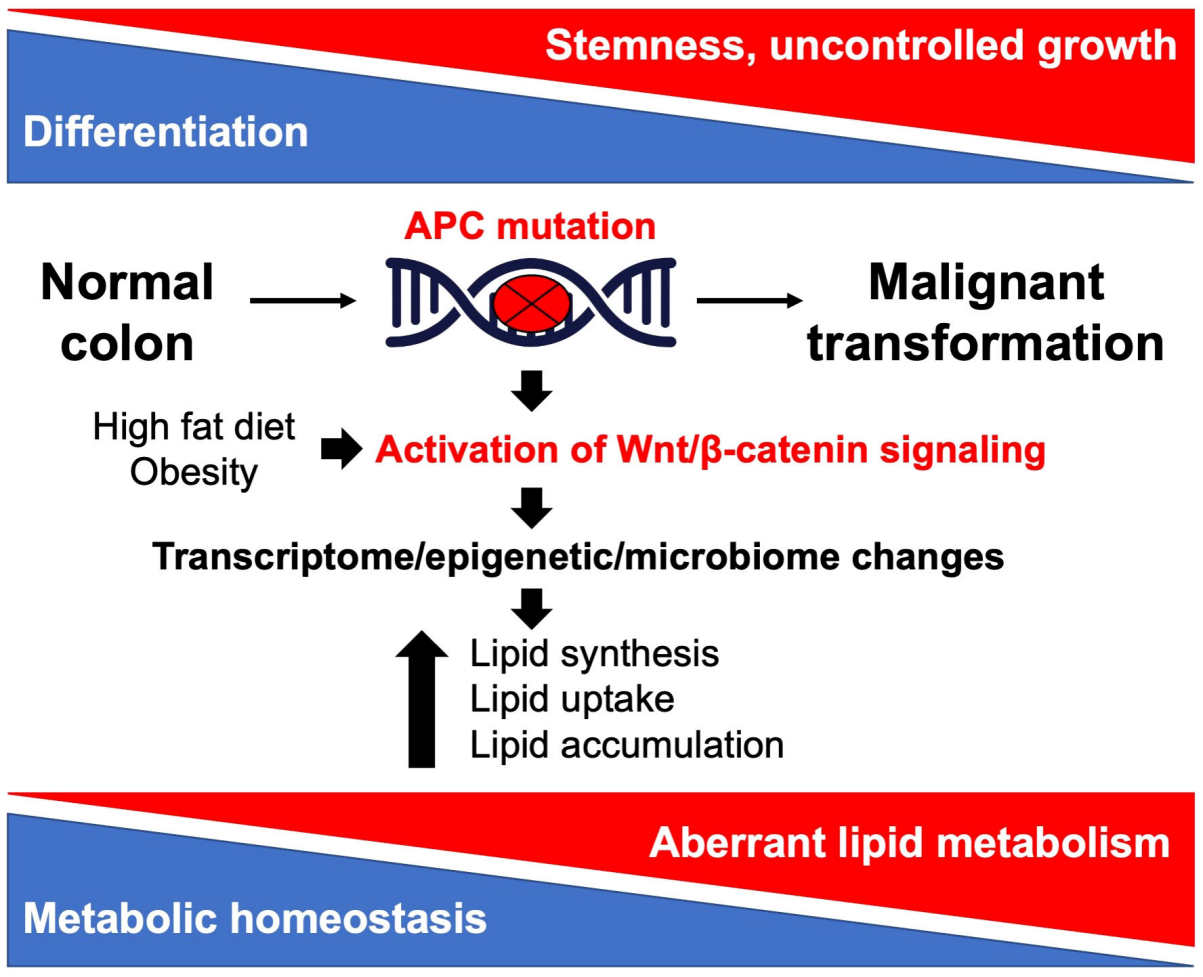
Illustration of the crosstalk between Apc/Wnt signaling and lipid metabolism in colorectal cancer.

**Figure 2:**
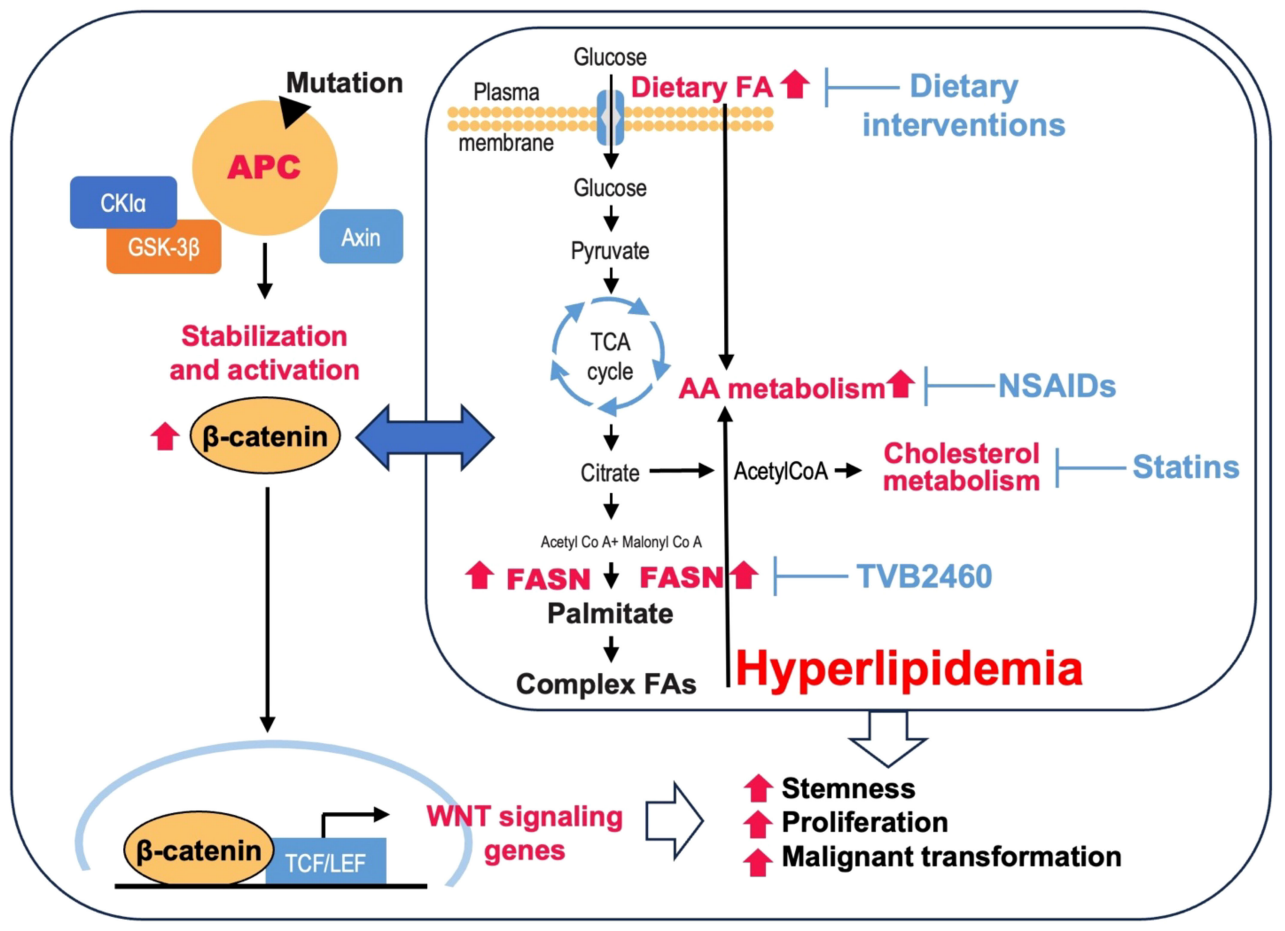
Potential lipid metabolism targets in *APC*-driven cancer. Loss of *APC* function due to mutations promotes translocation of β-catenin to the nucleus and increases transcription of Wnt target genes. Mutations in *APC* and an increase in Wnt signaling are associated with hyperlipidemia via upregulation of *de novo* lipid synthesis and an increase in dietary fatty acids (FA) in adenomas. Hyperlipidemia further promotes activation of Wnt signaling. Inhibition of *de novo* lipid synthesis using a novel FASN inhibitor TVB2640, cholesterol metabolism using statins, AA metabolism using NSAIDs, and dietary interventions are potential therapeutic approaches in *APC*-driven CRC.

As a common characteristic of cancer, altered lipid metabolism has been actively investigated as a potential target for therapeutic interventions (18). Dysregulated lipid metabolism presents a targetable metabolic vulnerability in CRC. Lipids are distributed throughout the cell and perform a variety of functions in signal transduction and membrane trafficking as well as a significant role in energy production and storage (19). Alterations in lipid metabolism can contribute to tumor initiation, progression, and metastasis at multiple levels by promoting malignant transformation, cell stemness, enhancing tumorigenic properties of cancer cells, and creating a permissive tumor environment for cancer growth and metastasis (20–22). Lipids can be synthesized *de novo* or obtained from diet (18). Both *de novo* lipid synthesis and dietary fatty acid uptake have been actively investigated as potential targets for therapeutic intervention in CRC. However, there is limited understanding of the crosstalk between the *APC*/Wnt signaling pathway, its downstream effectors, and lipid metabolism as well as how this crosstalk can contribute to CRC initiation, progression, and metastasis. This review summarizes and discusses the current understanding of the link between the mutations in *APC* gene/activation of Wnt signaling and upregulation of lipid metabolism (**Figure 1**) and how the interaction between these pathways can be targeted to improve the outcome of CRC (**Figure 2**; **Table 1**).

**Table 1:** The potential lipid metabolism targets for therapeutic intervention in APC-driven colorectal cancer.

Druggable lipid metabolism targets	Model	Drugs	References
Arachidonic acid pathway	Primary murine colon epithelial cells and macrophages, human CRC cells	NSAIDs	(23–25)
	*Apc* mouse models	NSAIDs	(26)
	Population studies	NSAIDs	(27–30)
Cholesterol metabolism	Human APC mutant and wild type cells and patient-derived xenografts	Statins	(31)
	*Apc* ^Min/+^; human cohort	Statins	(32)
	Population studies	Statins	(33)
*De novo* lipid synthesis (FASN)	*Apc* ^Min/+^	TVB-2640 (in clinical trial)	(34)

## 2 The APC/Wnt signaling pathway in CRC initiation and progression

The *APC* locus was initially identified based on its association with FAP (35–38). The molecular characterization of human CRC led to the understanding that mutations of the *APC* gene play a major role early in the development of CRC (39). Individuals with FAP inherit mutations in the *APC* gene that lead to formation of thousands of adenomatous polyps, resulting in 100% onset of CRC, typically before the age of 40 if left untreated (40). Furthermore, these individuals acquire biallelic *APC* mutations, with loss of function in one allele followed by an acquired mutation in the second allele of *APC* in the adenomas and adenocarcinomas that develop (41). These acquired biallelic *APC* mutations are recognized as an early and rate limiting step in most sporadic colorectal tumors and all FAP-associated colorectal tumors (13). The germline mutation type and location determine the severity of disease including colonic polyp burden and the presence of extra-colonic FAP manifestations (42). Subtle nonsense, deletion, or nucleotide insertion mutations contribute to biallelic mutations in *APC* (43). The “top–bottom” and “bottom–top” models are the proposed hypotheses pertaining to the cell of origin within the crypt–villus axis to initiate CRC. Preston et al. found that stem cells at the base of the intestinal crypts induce the initiation event of colorectal adenomas of both sporadic and FAP adenomas by stimulating initial growth via crypt fixation which spreads into adjacent crypts as a secondary event, thus supporting the bottom–top model (44). In contrast, abnormal patterns of proliferation and dysplastic epithelium observed at the tops of crypts in differentiated colonocytes in sporadic tumors suggest that the top–bottom model is responsible for CRC initiation (45, 46). Interestingly, Wnt activation, subsequent of *APC* or *CTNNBL1* mutation, has been identified as an important contributor to malignant transformation in both models (47).

*APC*-mediated activation of the Wnt signaling pathway is one of the essential early events in the development of CRC (48). The large multi-domain of APC contains various subunits which allow it to bind and interact with various proteins to perform numerous processes like cell migration, cell adhesion, proliferation, differentiation, chromosome segregation, and negative regulation of Wnt/β-catenin signaling (7). The most conserved domain is the armadillo repeat-domain (7). Studies have shown this domain contributes to cell migration and cell adhesion via its binding interactions with cellular molecules including IQ-motif-containing GTPase activation protein 1 (IQGAP1), PP2A, Asef, and KAP3 (49–52). Subsequently, the 15–20-residue domain and SAMP repeats play crucial roles in negative regulation of the Wnt/β-catenin pathway by facilitating the proteasomal degradation of β-catenin (7). Moreover, mutations in the *APC* gene usually create truncated gene products which lead to the loss of various β-catenin binding sites (20R), Axin interaction sites (SAMP), nuclear localization sequences, and a C-terminal basic region which mediates cytoskeletal interactions (14).

The Wnt signaling pathway plays a crucial role in homeostatic regulation of the epithelium in the intestine (13). Furthermore, this pathway is responsible for regulating differentiation along the crypt–villus axis (53). A major function of the *APC* gene is tumor suppression of the Wnt signaling pathway via controlling and destabilizing β-catenin turnover. In the absence of a Wnt signal, β-catenin is first phosphorylated by the destruction complex comprised of APC, glycogen synthase kinase 3β (GSK3β), Axin, and casein kinase 1 (CK1) (7). Subsequently, β-catenin is ubiquitinated by β*-*TrCP200, and finally targeted for proteasomal degradation. In this scenario, the T cell factor/lymphoid enhancer factor (TCF/LEF) in the nucleus binds to the co-repressor protein, Groucho, to suppress transcriptional activity of Wnt targeted genes (7). When a palmitoylated Wnt ligand binds to Frizzled (Fzd) and low-density lipoprotein receptor related protein (LRP) co-receptor, the downstream effects allow β-catenin to evade phosphorylation and enter the nucleus. Specifically, the receptors form a complex to activate the Dishevelled (Dsh) protein. Dsh and Axin bind together to facilitate the formation of Wnt/Fzd/LRP5/6 complex, which prevents formation of the destruction complex (7, 16). Consequently, β-catenin remains stabilized and accumulates in the cytoplasm before it translocates in the nucleus to bind to TCF/LEF. This binding interaction stimulates the transcription of Wnt target genes, such as cyclin D1, c-Myc, Lgr5, promoting proliferation, cell cycle, and stem-like properties, respectively (7, 16). Interestingly, a recent publication demonstrated that intestinal *APC* mutants outcompeted their wild type counterparts by secreting Notum, a Wnt antagonist/inhibitor, to decrease their stemness and promote differentiation (54). Furthermore, Notum has been identified as a cancer stem cell marker in CRC (55). In gastric cancer cells, Notum enhances stem-like properties by upregulating Sox2 via the PI3K/AKT pathway (56).

Physiological factors within the intestinal environment stimulate epithelial cells to move from the crypt to the villus. In the villus, these epithelial cells differentiate from immature progenitor cells to mature enterocytes in which they lose their ability to proliferate and divide. The optimum activity of the Wnt signaling pathway occurs at the crypts where the progenitor and stem cells are located, and their proliferative activity is the highest, thus, contributing to the initiation and progression of CRC (13).

## 3 Lipid metabolism in *Apc*-deficient mouse models

To investigate the significance of *APC* inactivation in initiation of CRC in animals, Moser et al. created *Apc* ^Min/+^, the first germline mutant mouse model of intestinal neoplasia. *Apc* ^Min/+^ carries an autosomal dominant loss of function mutation of the *Apc* gene generated by exposure to *N*-ethyl-*N*-nitrosourea, a highly potent mutagen (57). In this model, a nonsense mutation (TTG to TAG) at the 850^th^ codon of the *Apc* gene leads to a truncated APC protein and makes these mice prone to intestinal adenomas. *Apc* ^Min/+^ mice spontaneously develop more than 50 adenomas, on average, throughout the intestinal tract and rarely survive beyond 150 days of age due to anemia and intestinal obstruction (58). The severity of this phenotype decreases the progression of adenoma to invasive carcinoma. While *Apc* ^Min/+^mice have been used as a model of human *APC* mutation, these types of *APC* truncations are not common in inherited and sporadic human CRC (59). Human CRC mutations in *APC* typically result in truncation of the C-terminal half of the protein without affecting the first 20-amino acid repeat (60). To better recapitulate human disease, *Apc* ^1309^ and *Apc* ^1322T^ mouse models were developed to express truncated APC that retains the first 20-amino acid repeat. Both of these models develop polyps in the small intestine, but the polyps are more proximal than those in *Apc* ^Min/+^ mice (59). Interestingly, the hyperlipidemic state has been observed in both *Apc* ^Min/+^ and *Apc* ^1309^ mice, suggesting that *Apc*-driven carcinogenesis is associated with upregulation of lipid metabolism (61). This study shows that both *Apc* ^1309^ and wild-type (WT) mice have equally low serum levels of triglyceride (TG) and cholesterol at 6 weeks of age, but by 12 weeks of age the TG level increases 10-fold in *Apc* ^1309^ mice but not in their wild-type counterparts. Moreover, this study demonstrates that aged *Apc* ^1309^ mice have not only a high cholesterol level but also significantly increased centrilobular-restricted steatosis in the liver. Similar findings were observed in *Apc* ^Min/+^ mice at 15 weeks of age (61). Interestingly, another study shows that TG and total cholesterol uptake are inhibited in the intestine of *Apc* ^Min/+^ mice with age, and downregulation of β-oxidation, oxidative stress responses, and cholesterol absorption via the intestinal peroxisome proliferator-activated receptor α (PPARα) are associated with cachexia (62). Consistently, intracellular lipid changes induced by *Apc* gene mutations were shown in intestinal organoids established from either WT or mice with *Apc* mutations (Lgr5-EGFP-IRES-CreERT2*Apc* ^fl/fl^). Using a reversed-phase ultra-high-performance liquid chromatography mass spectrometry-based lipid profiling method, this study showed that the levels of phospholipids (e.g., PC(16:0/16:0), PC(18:1/20:0), PC(38:0), PC(18:1/22:1)), ceramides (e.g., Cer(d18:0/22:0), Cer(d42:0), Cer(d18:1/24:1)) and hexosylceramides (e.g., HexCer(d18:1/16:0), HexCer(d18:1/22:0)) were higher in *Apc* ^fl/fl^ organoids as compared to WT organoids. In contrast, the levels of sphingomyelins (e.g., SM(d18:1/14:0), SM(d18:1/16:0)) were lower in *Apc* ^fl/fl^ organoids as compared to WT mice. These results suggest that the loss of *Apc* in the organoids leads to alterations in lipid metabolism associated with an increase in intracellular PC, Cer, and HexCer levels (63).

In many human CRCs, Wnt signaling is hyperactivated due to genetic alterations of *APC*. The level of Wnt activation and its downstream signaling is dosage-dependent based on the number of β-catenin binding repeats that remain in the truncated APC protein (64). An interesting observation made in a recent study is that the level of oncogenic Wnt hyperactivation impacts the metabolic homeostasis, and the metabolism of Wnt‐high tumors in general relies more on mitochondrial metabolism than that of Wnt‐low tumors (65). This study highlights the complexity of the APC/Wnt/β*-*catenin pathway in different cell types in a context‐dependent manner.

In adipocytes, the canonical Wnt/β-catenin pathway regulates *de novo* lipogenesis and fatty acid monounsaturation (66). Further, it has been shown that β-catenin mediates the effects of Wnt signaling on lipid metabolism in part by regulating the transcription factors such as MLX interacting protein like *(MLXIPL)* and sterol regulatory element binding transcription factor 1 (*SREBF1)*. This study found that β-catenin deletion in mice promotes resistance to diet-induced obesity and it occurs via decrease in adipocyte hypertrophy and subsequent protection from metabolic dysfunction (66). Consistently, hepatocyte-associated canonical Wnt signaling is essential for the development of diet-induced fatty liver and obesity (67). β-catenin has also been shown to bind transcription factors involved in regulation of lipid metabolism including peroxisome proliferator-activated receptor γ (PPARγ), retinoid X receptor α (RXRα), and retinoic acid receptor (RAR) (68–70). Dysregulated Wnt signaling due to *Apc* mutation can also alter bile acid profiles and increase levels of tauro-β-muricholic acid and deoxycholic acid to drive malignant transformations in Lgr5-expressing (Lgr5^+^) cancer stem cells and promote a progression from adenoma to adenocarcinoma (71). Loss of phospholipid-remodeling enzyme lysophosphatidylcholine acyltransferase 3 (LPCAT3) or driving endogenous cholesterol synthesis through activation of sterol regulatory element-binding protein 2 (SREBP-2) in *Apc* ^Min/+^ mice markedly promotes intestinal tumor formation, suggesting the crucial importance of cholesterol synthesis and availability in regulation of APC-driven intestinal tumorigenesis (72). Importantly, mutations in *Apc* promote membrane rigidity and lipid raft stability via an increase in plasma membrane cholesterol, an essential component of the ordered membrane domains. The global transcriptome analyses suggest that the oncogenic APC-induced alterations in cholesterol homeostasis are partly mediated by changes in gene expression associated with cholesterol uptake, efflux, synthesis, intracellular trafficking and metabolism. Changes in plasma membrane lipid homeostasis and in organization of Wnt signaling plasma membrane domains driven by *Apc* loss and aberrant Wnt signaling lead to vital changes in stimulation of interactions between Wnt receptor and their effectors, thus, further promote oncogenic Wnt signaling and tumorigenesis (73). Besides the role of lipids in remodeling of cellular membranes to promote Wnt signaling, altered lipid metabolism may enhance Wnt signaling by lipidation of Wnt proteins (74). For example, protein-serine O-palmitoleoyltransferase, a key regulator of the Wnt signaling, mediates lipidation of Wnt proteins which is essential for their secretion and signaling activities (75). However, there is a limited data on characterization of Wnt protein lipidation and further studies are needed to understand it’s impact in early intestinal carcinogenesis.

Consistent with other studies showing the impact of *Apc* loss on lipid metabolism, our group has shown that development of adenomas in an *Apc*/VillinCre mouse model is driven by *de novo* lipid synthesis and heterozygous deletion of FASN, a key enzyme of lipid biosynthesis that significantly increases mouse survival and decreases the number of adenomas formed (34). High FASN expression during *Apc*-driven colon carcinogenesis is associated with enrichment of genes associated with Wnt pathway, FA metabolism, and steroid biosynthesis. Interestingly, heterozygous deletion of FASN in intestinal epithelial cells leads to a significant decrease in the levels of several diglyceride species (C16:1:20:0-DAG; Di-C14-DAG; C14:0:18:0-DAG; C16:1:18:0-DAG; C18:0:18:1-DAG) in mouse adenoma tissues but does not affect the total levels of free fatty acid and sphingolipid species (34). Interestingly, pharmacological inhibition of FASN and lipid biosynthesis disrupts lipid rafts architecture, decreases protein lipidation, and thus, inhibits Wnt/β*-*catenin signal transduction (76), suggesting these might be the potential mechanisms behind FASN knockout on polyp formation in transgenic animals.

In summary, *Apc* inactivation leads to significant alteration in intestinal lipid metabolism including upregulation of *de novo* lipid synthesis and cholesterol synthesis, thus, contributing to stemness, cellular proliferation, survival, lipid raft stability, and support of prooncogenic signaling.

## 4 Contribution of dietary fatty acids and obesity to *Apc*-driven carcinogenesis *in vivo*

It has been shown that a high-fat diet and obesity accelerate the development of intestinal adenomas in mice with mutation or deletion of the *Apc* gene (77). This study shows that high-fat-diet-induced inflammation from specific dietary fatty acids, rather than obesity or metabolic status, is associated with increased *Apc*-driven intestinal polyposis (77). Consistently, another study found that when a missense mutation that results in premature termination of the intracellular domain of the long form of the leptin receptor (Lepr^db/db^)—which results in the phenotype of obesity and diabetes mellitus—is introduced into the *Apc* ^1638N/+^ background, it induces the formation of not only intestinal but also gastric and colonic tumors (78). The potential mechanisms that drive adenoma formation in C57BL/KsJ-db/db-*Apc* ^Min/+^ mice are increased expression of insulin-like growth factors (IGFs), as well as hyperlipidemia and hyperinsulinemia (79). Another proposed mechanism is that diet-induced obesity increases the number and function of Lgr5+ ISCs, while also promoting stemness and tumorigenicity of progenitor cells after inactivation of *Apc* (80). Furthermore, it has been shown that a high-fat diet increases oxidative stress and alters the expression of intestinal gap junction proteins, thereby accelerating membrane permeability and endotoxemia, increasing inflammation, and thus, promoting intestinal tumorigenesis in *Apc* ^Min/+^ mice (81). Using ChIP-seq and RNA-seq approaches in *Apc* ^Min/+^ mice, authors of a recent publication demonstrated that keeping mice on a high-fat diet for only three days resulted in epigenetic and transcriptomic changes in lipid metabolic pathways and accelerated tumor growth as compared to mice kept on a low-fat diet. In addition, a high-fat diet has been shown to suppress the activity of pathways related to the immune system, suggesting that an increase in lipid uptake and metabolism provides a more permissive immunologic environment that favors tumor progression (82).

In addition to the effects of high-fat diet on transcriptome, epigenome, and metabolism, it promotes CRC development dependent on gut microbiota (83). To support this finding, paired microbiome and metabolome analyses show that high-fat diet is a dominant determinant of cecal microbiome and metabolome in *Apc* ^Min/+^ mice and implicates microbially conjugated bile acids as potential drivers of polyposis and CRC progression (84).

One of the potential mechanisms of how high-fat diet and obesity promote intestinal carcinogenesis is through increased activity of the pro-tumorigenic Wnt signaling. The associations of obesity, microbiome, inflammation, and Wnt signaling were examined in *Apc* ^+/1638N^ mice whose obesity was induced by either diet-modified or genetically modified obesity. Studies of *Apc* ^+/1638N^Lepr^+/+^ fed a low-fat diet (10% kcal fat), *Apc* ^+/1638N^Lepr^+/+^ fed a high-fat diet (60% kcal fat), and *Apc* ^+/1638N^ Lepr^db/db^ fed a low-fat diet show that the elevated inflammation and changes in microbiome were associated with altered expression of genes associated with oncogenic Wnt signaling and enhanced tumorigenesis (85).

Another mechanism of how lipids can promote colorectal tumorigenesis is through loss of free fatty acid receptor 2 (FFAR2), which is expressed in colon and can be activated by short-chain fatty acids. Loss of FFAR2 promotes the development of colon adenoma in *Apc* ^Min/+^ mice and enhances long-chain fatty acid β-oxidation and bile acid metabolism (86). These changes in lipid metabolism are associated with significant changes in gut microbiota and an increase in relative abundance of *Flavobacteriaceae* and *Verrucomicrobiaceae* in the *Apc* ^Min/+^-*Ffar2* ^-/-^ mice compared to the *Apc* ^Min/+^ mice, which may contribute to colon adenoma development in *Ffar* -deficient mice (86). The crucial role of *Apc* inactivation and dysregulation of Wnt/β-catenin signaling in obesity is shown in a study demonstrating that inactivation of *Pten* in Lgr5+ ISCs, whether alone or in combination with obesity, is insufficient to drive adenoma formation in mice, but combination of *Pten* deletion with *Apc* loss increases tumor burden and worsens survival (87).

In summary, both high-fat diet and obesity promote *Apc*-driven carcinogenesis via multiple mechanisms involving changes in transcriptome, metabolome, and microbiome, thus promoting inflammation, stemness, and altering immune responses.

## 5 Mutant *APC* and lipid metabolism in human studies

Lipid metabolic pathways that are affected in CRC cells include lipid uptake, lipid synthesis, desaturation, elongation, and mitochondrial oxidation of fatty acids (88). Human lipid profiles depend on many factors including polyp/tumor heterogenicity, diet, and environmental exposures. Therefore, currently available data on CRC lipidome are broad and fraught with inconsistencies. Here, we review several studies performed on FAP/polyp tissues to discuss alterations in lipid metabolism occurring due to *APC* mutations.

Similar to data reported from the *Apc*-driven mouse models, studies in FAP show that intestinal polyps exhibit a hyperlipidemic state. Analysis of 28 FAP patients from the National Cancer Center Hospital in Japan shows that the overall prevalence of hyperlipidemia in the patients was 57.7% (15/26) and the prevalence of hypercholesterolemia (220–296 mg/dl) was 53.3% (8/15) (89). Analysis of serum metabolites from 30 FAP patients and 34 healthy individuals detected and quantified using ultra performance liquid chromatography and tandem mass spectrometry (UPLC−MS/MS) demonstrated an increase in metabolites associated with fatty acid metabolism, cholesterol metabolism, bile acid biosynthesis, and bile secretion. In particular, the level of arachidonic acid (AA) was significantly higher in the serum of FAP patients compared with healthy controls (90). Another study reported the fatty acid composition in serum of 38 colectomized FAP patients and 160 healthy individuals. It showed that the levels of linoleic and α-linolenic acid were higher in healthy subjects (difference: 3.96, 95% CI: 2.87–5.04 and 0.06, 95% CI: 0.04–0.08, respectively), and the AA and docosahexaenoic acid levels were lower in healthy individuals (difference: −3.70, 95% CI: −4.35 to −3.06 and −5.26, 95% CI: −6.25 to −4.28, respectively) as compared to the FAP patients (all *p* ≤ 0.0001) (91). Consistent with previous reports, a study using multi-omics profiling of 135 normal mucosal, benign, and dysplastic polyps and adenocarcinoma samples from six FAP patients showed AA pathway upregulation in benign polyps relative to FAP mucosa. Moreover, this study showed depletion of TG stores suggesting that this lipid class is a key energy source for polyp formation and growth (92). Utilizing human induced pluripotent stem cells (iPSC) from healthy individuals or from patients with FAP, a study showed that *APC* heterozygosity is responsible for major changes in the transcriptional profile of the cells and key signaling pathways associated with pro-tumorigenic signaling, including alterations in lipid metabolism and PPAR signaling pathway (93). Together, these studies show that hyperlipidemia and altered lipid metabolism are frequently observed in FAP, suggesting its possible link to development of CRC.

To further support the role of lipid metabolism in malignant transformation and CRC, another study investigated CRC lipidome for putative tumor-specific alterations through analysis of three independent retrospective patient cohorts to show that signatures of sphingomyelin and TG species are significantly different in cancerous versus non-diseased tissue. The results of this study demonstrated an increase in the levels of sphingomyelin species with 32–34 carbons and a decrease in the levels of sphingomyelin species with more than 34 carbons in tumor samples. In contrast, the levels of sphingosine-based Cer with longer chains (C24:0–C26:0) were increased and the levels of sphingosine-based Cer with shorter (<C22:0) chains were decreased in tumor samples. For TGs, species with <53 carbons were decreased, while polyunsaturated TGs with 56 carbons were increased in tumor samples. Moreover, the expression of lipogenic enzymes was significantly upregulated in tumor tissue, and FASN gene expression is prognostically detrimental in CRC (94). Even though this study did not assess lipidomic changes in human tumor tissues according to the *APC* status and staging, the additional quantitative lipidome analysis was performed on adenomas from *Apc* ^1638N^ mice to test whether deregulation of TG species is conserved in mice. Consistent with human data, TG containing poly-unsaturated acyl chains (TG 56:4, TG 56:5, TG 56:6) had a significantly higher abundance in adenomas, whereas shorter and less unsaturated TGs were lower (94). Similarly, FASN was significantly upregulated in mouse adenomas (94), which is consistent with data from our group and suggest that upregulation of FASN is an early event in *Apc*-driven CRC (34).

In summary, the majority of studies using human tissues demonstrate upregulation of lipid metabolism including *de novo* lipogenesis and AA metabolism in polyps driven by mutations in *APC*, suggesting that these pathways can be the major contributors to CRC development and metabolic vulnerabilities during CRC initiation.

## 6 Discussion: clinical implications and therapeutic strategies based on current knowledge of crosstalk between loss of *APC* and lipid metabolism

Research by Hans Clevers and colleagues shows that *Apc* restoration triggers differentiation and restores crypt homeostasis which drive tumor regression in CRC, thus validating the Wnt pathway as an effective therapeutic target in CRC (95). Multiple strategies that target Wnt signaling have been developed, from small molecules to blocking antibodies, and peptide agonists and antagonists (96). However, one of the major challenges of targeting Wnt signaling downstream of mutant *APC* is significant toxicity since this pathway plays a crucial role in normal development, stem cell maintenance, tissue regeneration, and adult tissue homeostasis (95, 96). In addition, the crosstalk of Wnt signaling with other signaling pathways further challenges the development of effective therapeutics.

Multiple studies suggest that lipid metabolism may be a metabolic vulnerability in CRC, including *APC*-driven CRC (18, 34, 88, 94). The preclinical studies in *Apc* mouse models demonstrate that inactivation of *Apc* is associated with upregulation of fatty acid metabolism and contributes to proliferation of adenomas (34, 61, 63). More importantly, analysis of lipid metabolism in human polyps and tumor tissues may provide better insight into metabolic disturbances that contribute to carcinogenesis and provide new therapeutic strategies for identification, prevention, and therapy of this disease. Upregulation of the AA pathway occurs early in hyperplasia (90, 91) and can activate tumor-related gene expression and signaling pathways (97). This pathway is targeted by non-steroidal anti-inflammatory drugs (NSAIDs), a common preventative treatment for FAP patients. Cyclooxygeneses (COX-1 and COX-2), key enzymes involved in synthesis of prostaglandins from arachidonic acid, are targets of common NSAIDs. Several preclinical studies support the role of the AA pathway in malignant transformation. An increase in production of 4-hydroxy-2-nonenal (4-HNE), a DNA mutagen and an autochthonous mitotic spindle poison, produced by peroxidation of ω6 polyunsaturated fatty acids, and upregulation of COX-2 in primary colon cells by commensal infected microphages have been reported as CRC initiation contributing factors (23–25). Inactivation of COX-1 and COX-2 have been shown to significantly reduce polyp numbers in multiple animal models (26). Furthermore, knockout mutation for prostaglandin E2 receptor EP2 introduced into *Apc* ^Δ716^ mice results in a reduced number and size of intestinal polyps (98). Importantly, the clinical trial with celecoxib, a selective COX-2 inhibitor, in patients with FAP showed a significant reduction in the number of colorectal polyps (99). Furthermore, a prospective cohort study by Tangrea et al. found a significant reduction in adenoma recurrence in NSAID users, particularly in advanced polyps (28, 30). Interestingly, multiple studies report differences in the gut microbiome and metabolome in patients using NSAIDs as compared to those not using NSAIDs, suggesting that protective anti-inflammatory effects of NSAIDs on polyp formation are potentially mediated by intestinal flora and microbe-derived metabolites (27, 29).

The recent study using a meta-analysis to assess the impact of NSAIDs on colorectal polyp burden in patients with FAP concluded that a short-term use of NSAIDs reduces polyp number and polyp size but with low to very low certainty of evidence (100). Furthermore, evidence supports an association between aspirin use and reduced CRC-related mortality in subpopulations, including tumors overexpressing COX-2 and proximal CRCs (30). Therefore, additional studies are needed to better elucidate the association between use of non-aspirin NSAIDs and CRC prevention low-risk, elderly, and other populations.

There is strong evidence that dietary factors can affect the risk and development of CRC. Several preclinical and human observational studies show the benefits of fish oil, which is rich in two omega-3 fatty acids (EPA and DHA) (101). Results of the Seafood Polyp Prevention Trial, in which 2 g EPA-free fatty acid (FFA) per day (either as FFA or triglyceride), 300 mg aspirin per day, both, or placebo were administered for 12 months, showed no effect on polyp formation; however, it showed a significant decrease in the recurrence of some subtypes of adenomas with both EPA and aspirin (102). In another study, daily supplementation with marine ω-3 fatty acids (1 g) did not reduce the risk of CRC development in the general US population; however, potential benefits were observed in individuals with low plasma levels of ω-3 and in African Americans (103). These studies demonstrate that further research, including research in different racial and ethnics groups, is needed regarding potential therapeutic and dietary intervention to prevent and target APC-driven CRC.

Upregulation of FASN and lipid synthesis is critically important in *Apc*-driven CRC initiation as it orchestrates changes in the transcriptome, proteome, and metabolic pathways consistent with increased cellular proliferation, ATP production, anabolism, and adenoma formation (34). Therefore, inhibition of FASN should be further investigated as a potential preventive strategy or early-stage treatment for CRC. Currently, a novel FASN inhibitor, TVB-2640, is being tested in several Phase I-II oncology clinical trials (104, 105); thus, providing the opportunity to test inhibition of *de novo* lipogenesis as a preventive therapeutic approach for CRC.

Targeting cholesterol synthesis may be another potential strategy for CRC. Statin use is associated with a lower risk of incident CRC, CRC-related mortality, and all-cause mortality (33). Several studies have demonstrated that statin use exhibits antitumor effects in CRC. Mechanisms that may be responsible for the antitumor effect of statins include induction of apoptosis and inhibition of cell proliferation, angiogenesis, immune response, and metastatic capacity (106). Interestingly, a recent study shows that treatment with statins leads to a significantly greater inhibition of cell viability and expression of Wnt target in the *APC* mutant cell lines and patient-derived xenograft models as compared to wild-type *APC* cells, supporting the use of statins in *APC*-driven CRC (31). Recent evidence also suggests that the protective effects of statins can be associated with reduced dysbiosis in the gut microbiome. It has been shown that atorvastatin treatment leads to an increase in *Lactobacillus reuteri* and microbial tryptophan availability, thus eliciting anti-tumorigenic effects via the tryptophan catabolite, indole-3-lactic acid (ILA). The study suggests that interventions with *Lactobacillus reuteri* or ILA could complement current preventive strategies for CRC (32).

In summary, lipid metabolism is an attractive therapeutic target for CRC initiation, but more studies are needed to better elucidate the mechanistic crosstalk between activation of oncogenic pathways such as APC/Wnt/β-catenin and lipids. Finally, consideration of racial, gender, and dietary differences is important to develop efficacious therapeutic approaches to target lipid metabolism in CRC.

## Author contributions

CK: Writing – original draft, Writing – review & editing, Conceptualization. YZ: Supervision, Funding acquisition, Writing – review & editing, Writing – original draft, Conceptualization, Validation.
